# Acknowledgement to Reviewers of *Biomimetics* in 2017

**DOI:** 10.3390/biomimetics3010001

**Published:** 2018-01-15

**Authors:** Biomimetics Editorial Office

**Affiliations:** MDPI AG, St. Alban-Anlage 66, 4052 Basel, Switzerland

Peer review is an essential part in the publication process, ensuring that Journal maintains high quality standards for its published papers. In 2017, a total of 23 peer-reviewed papers were published in the journal. Thanks to the cooperation of our reviewers, the median time to first decision was 33 days and the median time to publication was 63 days. The editors would like to express their sincere gratitude to the following reviewers for their time and dedication in 2017:

Aldred, NicholasLao, Ka UnAljabali, AlaaLee, BruceBertinetti, LucaMessersmith, PhillipBettinger, ChristopherMilford, MichaelBindschedler, SaskiaMustoe, George E.Borges, JoãoPalumbo, FabioBreydo, LeonidParisi, CristianBumgardner, Joel D.Reisch, AndreasCarmagnola, IreneRuggeri, FrancescoCicoira, FabioRusso, NinoCirera, JordiScheiner, SteveDel Real, Juan CarlosSchroeder, GrzegorzDhinojwala, AliSlominski, AndrzejDickinson, GarySobolewski, Andrzej L.Drack, ManfredSolano, FranciscoEllinas, KosmasStone, KariFalentin-Daudré, CélineSturm, ElenaGarcía-Ruiz, Juan ManuelTaboryski, RafaelGerdon, ArenTaubert, AndreasHarbottle, MikeTavares, AnthonyHess, Dennis W.Thrivikraman, GreeshmaHong, SeonkiVenanzi, MarianoKeil, Matthias S.Webb, BarbaraKimura, ShunsakuWysokowski, MarcinKremer, Barbara
Ladavière, Catherine


